# Spatial Analysis of Severe Fever with Thrombocytopenia Syndrome Virus in China Using a Geographically Weighted Logistic Regression Model

**DOI:** 10.3390/ijerph13111125

**Published:** 2016-11-11

**Authors:** Liang Wu, Fei Deng, Zhong Xie, Sheng Hu, Shu Shen, Junming Shi, Dan Liu

**Affiliations:** 1 School of Information Engineering, China University of Geosciences, Wuhan 430074, China; wuliang@cug.edu.cn (L.W.); xiezhong@cug.edu.cn (Z.X.); hsh1n1@163.com (S.H.); 2 National Engineering Research Center for GIS, Wuhan 430074, China; 3 State Key Laboratory of Virology, Wuhan Institute of Virology, Chinese Academy of Sciences, Wuhan 430071, China; df@wh.iov.cn (F.D.); shenshu@wh.iov.cn (S.S.); sjm19901002@sina.com (J.S.); 4 School of Medicine, Wuhan University of Science and Technology, Wuhan 430081, China

**Keywords:** SFTSV, GWLR, spatial analysis, public health

## Abstract

Severe fever with thrombocytopenia syndrome (SFTS) is caused by severe fever with thrombocytopenia syndrome virus (SFTSV), which has had a serious impact on public health in parts of Asia. There is no specific antiviral drug or vaccine for SFTSV and, therefore, it is important to determine the factors that influence the occurrence of SFTSV infections. This study aimed to explore the spatial associations between SFTSV infections and several potential determinants, and to predict the high-risk areas in mainland China. The analysis was carried out at the level of provinces in mainland China. The potential explanatory variables that were investigated consisted of meteorological factors (average temperature, average monthly precipitation and average relative humidity), the average proportion of rural population and the average proportion of primary industries over three years (2010–2012). We constructed a geographically weighted logistic regression (GWLR) model in order to explore the associations between the selected variables and confirmed cases of SFTSV. The study showed that: (1) meteorological factors have a strong influence on the SFTSV cover; (2) a GWLR model is suitable for exploring SFTSV cover in mainland China; (3) our findings can be used for predicting high-risk areas and highlighting when meteorological factors pose a risk in order to aid in the implementation of public health strategies.

## 1. Introduction

Severe fever with thrombocytopenia syndrome (SFTS) is an emerging infectious disease with a fatality rate of 12%. It was first reported in 2009 in the rural regions of the provinces of Hubei and Henan in central China. The clinical symptoms of SFTS are non-specific and include high fever (temperature of ≥38 °C), thrombocytopenia (platelet count of <100,000/mm^3^), leukocytopenia, multi-organ dysfunction and hemorrhagic tendency [1,2,3]. SFTS has been reported not only in at least 13 provinces in the central, eastern and northern regions of China [4], but also in North Korea [5], South Korea [6] and Japan [7]. SFTSV has spread wider and wider, posing an increasingly significant threat to the global health. 

The majority of cases have been reported to occur in rural areas from April to July [8]. A novel phlebovirus of the family *Bunyaviridae* was identified as the causative agent of SFTS in 2010, and it was named severe fever with thrombocytopenia syndrome virus (SFTSV) or Huaiyangshan virus [1,9]. At the end of 2012, it was confirmed that SFTSV has been associated with SFTS in seven provinces in the central, eastern and northern regions of China, including the provinces of Hubei [10], Henan [11], Jiangsu [12,13], Liaoning [14], Anhui [15], Shandong [16] and Zhejiang [17].

The epidemiology, clinical signs and pathogenesis of SFTS have been widely studied [18,19,20,21,22], and over 200 individual nucleotide sequences of SFTSV have been submitted to GenBank. However, the transmission of SFTSV remains an important and yet unexplained process. A previous study suggested that SFTSV is an arbovirus that can be transmitted by *Haemaphysalis longicornis*, a species of tick [1]. Humans can become infected through tick bites or by contact with infected blood or bloody secretions from humans or livestock [23,24]. Moreover, many studies showed that tick-borne diseases like Lyme disease, rickettsiosis and tick-borne encephalitis have a strong association with environmental factors [25,26,27]. Considering that the majority of patients of confirmed SFTS cases lived in rural areas and were employed in agriculture [8,18], we hypothesize SFTS might be influenced by environmental factors and anthropogenic factors. Our study has two major objectives: (1) to identify the factors of SFTSV based on data from seven provinces with SFTSV cover and other provinces that had no reported cases; (2) to predict high-risk areas in China.

SFTSV cover means positive provinces or confirmed areas. The provinces experienced cases of SFTS that tested positive for SFTSV in patients were defined as SFTSV cover or confirmed areas of SFTSV. Furthermore, it might be difficult to distinguish SFTSV cover from reported SFTSV. Some provinces have even reported some cases with symptoms similar to SFTS, but the medics were unable to extract the SFTS virus from the patients. Therefore, these provinces were defined as SFTSV reported but not so-called SFTSV cover.

In this study, we estimated a geographically weighted logistic regression (GWLR) model and explore the spatial associations of SFTSV with the following potential determinants: meteorological factors (average temperature (AT), average monthly precipitation (AMP) and average relative humidity (ARH)), the average proportion of rural population (APRP) and the average proportion of primary industries (APPI).

## 2. Materials and Methods

### 2.1. Data Collection

SFTSV is a negative-sense RNA virus that contains three single-stranded RNA genome segments designated as large (L), medium (M) and small (S). All the available SFTSV data, including sequences and location information, from 2010 to 2012 were obtained from the database of the National Center for Biotechnology Information (NCBI) in the USA, which provides access to biomedical and genomic information.

The meteorological data were obtained from the weather website Reliable Prognosis 5 (rp5) (http://rp5.ru). This website, authorized by the Russian Federal Service for Hydrometeorology and Environmental Monitoring, allows creating and maintaining databases in the field of hydrometeorology and related fields. The meteorological data included the provincial capitals’ weather data from April to July of 2010, 2011 and 2012 concerning 31 provinces in the whole of China. We collected the weather data of provincial capitals representing the weather of corresponding provinces. The average values of three years of the weather data of provincial capitals were employed for analysis. The AT, AMP and ARH were assessed.

Anthropogenic factors were also considered. SFTS case was first reported in rural regions, and the lifecycle and transmission characteristics of ticks related to agricultural works of human beings. Therefore, we obtained data on the proportion of rural population and of primary industries in each of the provinces. The rural population data and industrial data from 2010 to 2012 were obtained from the National Bureau of Statistics of the People’s Republic of China [28,29,30]. The APRP and APPI were assessed. The units of five variables are presented in Table 1.

The mapping data from China were obtained from DIVA-GIS. GCS-WGS-1984 and a Lambert conformal conic projection for Asia were used to determine the geographic coordinate system and projection coordinate system, respectively.

**Figure 1:**
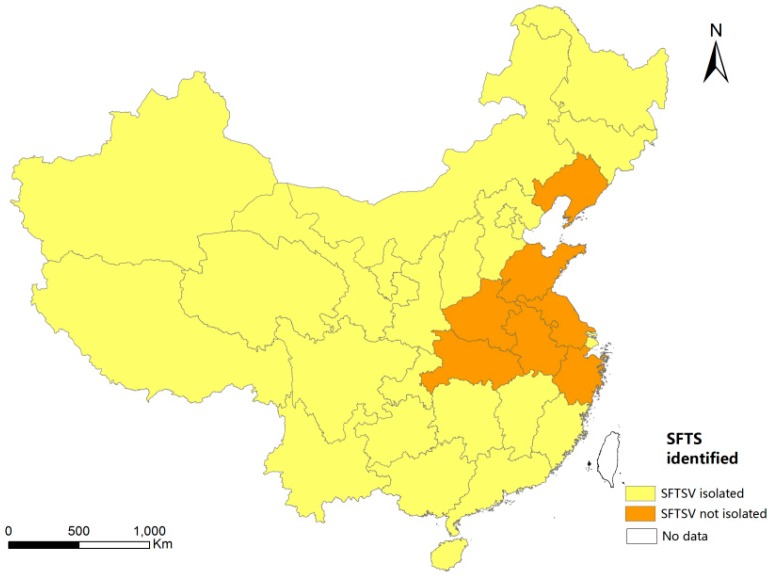
Geographical distribution of SFTSV in mainland China from 2010 to 2012. SFTSV = severe fever with thrombocytopenia syndrome virus.

We obtained information on 263 SFTSV sequences of viruses that had been isolated from patients from 2010 to 2012 and registered in the NCBI database; each sequence came with location information (i.e., information on the province where the virus was isolated). Therefore, we could easily determine whether SFTSV had been isolated in each province in China (except for the province of Taiwan, for which there was no data). Each province was assigned a value of 1 if SFTSV had been isolated from patients or 0 if SFTSV had not been isolated (Figure 1).

### 2.2. Modeling Methods

**Figure 2:**
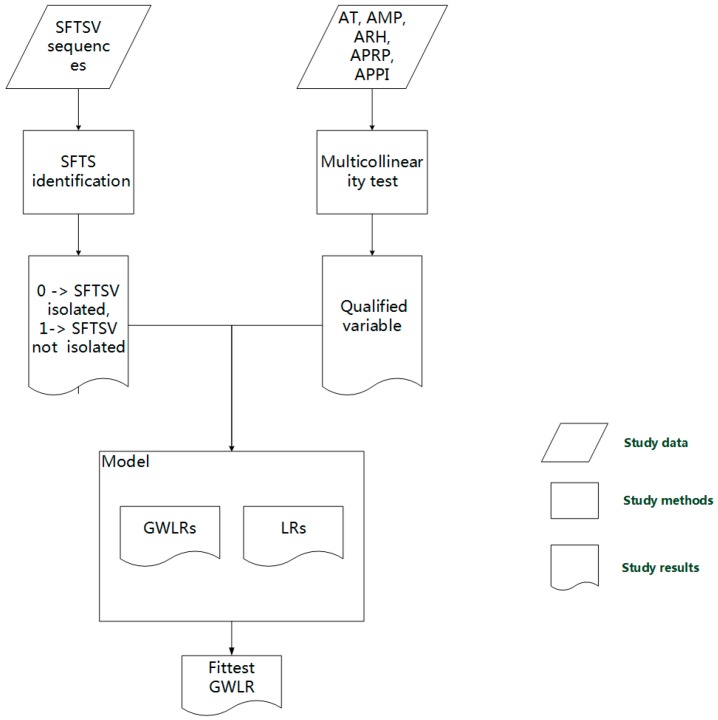
Study flow chart. AMP = average monthly precipitation; APPI = average proportion of primary industries; SFTS = severe fever with thrombocytopenia syndrome; APRP = average proportion of rural population; ARH = average relative humidity; AT = average temperature; GWLR = geographically weighted logistic regression; LR = logistic regression; SFTSV = severe fever with thrombocytopenia syndrome virus.

A GWLR model [31] (a spatial model) was estimated in order to explore the associations between various explanatory variables and SFTSV cover (Figure 2).

#### 2.2.1. Geographically Weighted Logistic Regression Model

In geographical spatial analysis, n sets of data represent the sampled data acquired from n different locations. The orthodox global regression model (e.g., ordinary least squares, OLS model) assumes that coefficients of regression model are not relevant to the geographical locations of sampled data, i.e., coefficients keep consistent in the whole study area [32]. But in practical applications, coefficients of regression model are always mutative in varying locations. If we still adopted the above model, the calculated coefficients would be the average value of the whole study area, which would not reflect the true spatial or distributed features. Aiming at the spatial heterogeneity, i.e., when the nature and significance of relationships between variables differs from location to location [33], a specifically designed technique, geographically weighted regression (GWR), is commonly used [34,35,36]. 

The logistic regression (LR) model is also a kind of global regression model, but different from the OLS model [37]. Unlike the continuous variable in the OLS model, the dependent variable in the LR model is discrete. For example, the relationship between the binomial dependent variable (the value of the variable is binomial, like 0 or 1, meaning yes or no respectively) and explanatory variables (discrete or continuous) would be fitted easily using the LR model, but the wrong results would be obtained if we took binomial variables in the OLS model.

The GWLR model combined a logistic regression model and a geographically weighted regression model and thereby extended the LR model by taking into account spatial heterogeneity [31]. In this type of model, the structure is considered non-stationary, meaning that the relationship between the binomial dependent variable (the value of the variable is 0 or 1 only) and the continuous explanatory variables (AT, AMP, ARH, APRP and APPI) varies across different locations. Continuous explanatory variables are the mean value of AT, AMP, ARH, APRP and APPI in three different years. Each location has its own specific estimated regression coefficients. The GWLR model is expressed as (Equation (1) [31]:

(1) $$\begin{array}{ll} \mathrm{logit}\left(p_{i}\right) & =\log\left(\frac{p_{i}}{1-p_{i}}\right)\\ & =\beta_{0i}\left(u_{i},v_{i}\right)+\beta_{1i}\left(u_{i},v_{i}\right)\times {\mathrm{AT}}_{i}+\beta_{2i}\left(u_{i},v_{i}\right)\times {\mathrm{AMP}}_{i}+\beta_{3i}\left(u_{i},v_{i}\right)\\ & \times {\mathrm{ARH}}_{i}+\beta_{4i}\left(u_{i},v_{i}\right)\times {\mathrm{APP}}_{i}+\beta_{5i}\left(u_{i},v_{i}\right)\times {\mathrm{API}}_{i} \end{array}$$

where *i* represents the location *i*, $\left(u_{i},v_{i}\right)$ are the coordinates of region *i* (which represents the provincial capital of province *i*), $p_{i}$ is the probability of SFTSV cover at region *i*, $\beta_{0i}\left(u_{i},v_{i}\right)$ represents the GWLR intercept coefficients associated with $\left(u_{i},v_{i}\right)$, while $\beta_{ji}\left(u_{i},v_{i}\right)$ (*j* ∈ {1, 2, 3, 4, 5}) represents the GWLR coefficients for AT, AMP, ARH, APRP and APPI at region *i*. We first drew a circle of a given radius called the kernel bandwidth at location *i*, and we then computed a set of spatial weights between location *i* and its neighbors within the circle according to distance. Finally, the model coefficients were estimated using weighted least-squares regression [38]. In this study, the bandwidth was determined using the minimum Akaike information criterion (AIC) value [39].

#### 2.2.2. Model Evaluation

When two or more variables convey the same information, these variables are said to exhibit multicollinearity (or collinearity), and the results are usually unreliable [40,41]. Multicollinearity can result in an over-counting type of bias and an unstable model. Therefore, multicollinearity should be assessed in order to reduce errors. Generally, multicollinearity between variables is explored by calculating a matrix of correlation coefficients or by computing relevant indictors, such as the tolerance coefficient or the variance inflation factor (VIF) [42,43] (and a variable is excluded if its score is larger than that experienced) [32]. In this study, the VIF of each explanatory variable and AUC values was calculated using Stata 14 software (StataCorp LP, College Station, TX, USA). Subsequently, we used the GWR 4.0 software (Tomoki Nakaya, Ritsumeikan University, Kyoto, Japan) [31,44] for fitting the GWLR model and compared the GWLR models based on the AIC. The model with the minimum AIC value was determined to be the most suitable model for the data.

We determined the probability of SFTSV cover at each location. The area under the relative operating characteristic curve (AUC) [45,46] was assessed in order to explore the model fit. The AUC represents how well the model distinguishes between the presence and absence of SFTSV. A larger AUC indicates a more suitable model. The value of the AUC ranges from 0 to 1.0, with 0 indicating perfect misclassification, 0.5 indicating complete randomness and 1.0 indicating perfect classification. Models with AUC values of 0.5–0.7 are considered to be poor, those with AUC values of 0.7–0.9 are considered to show moderate discrimination, and those with AUC values of 0.9–1.0 are considered to be good [38,45]. The AUC values were computed using a post-estimation function.

## 3. Results

### 3.1. Spatial Distribution of Potential Determinants of SFTSV in Mainland China

**Figure 3:**
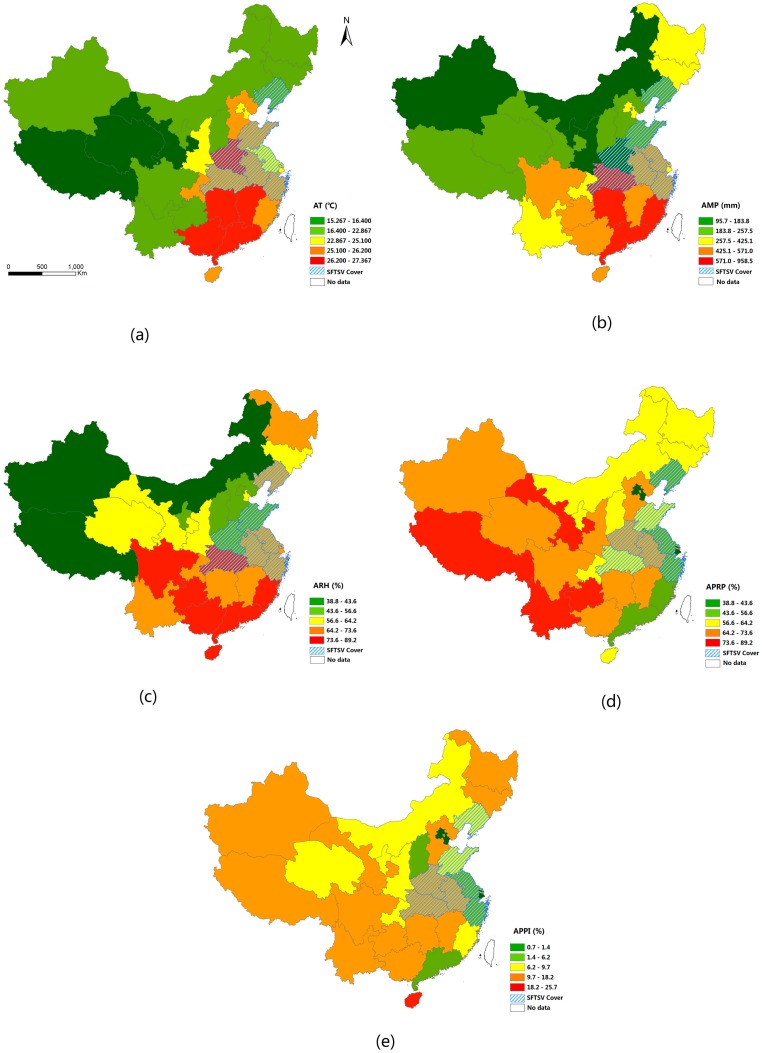
Spatial distribution of the five potential determinants of SFTSV cover: (**a**) Average temperature (AT) from 2010 to 2012; (**b**) Average monthly precipitation (AMP) from 2010 to 2012; (**c**) Average relative humidity (ARH) from 2010 to 2012; (**d**) Average proportion of rural population (APRP) from 2010 to 2012; and (**e**) Average proportion of primary industries (APPI) from 2010 to 2012.

Descriptive statistics for the meteorological data, the rural population data and the industrial data are presented in Figure 3. With regard to climate, the AT, AMP and ARH were higher in southeast China than in northwest China. With regard to the APRP, the proportion gradually increased from the east coast to inland areas. With regard to the APPI, there was no clear pattern across the country, and the province of Hainan had the highest proportion.

### 3.2. Frequency Analysis of Confirmed Cases of SFTSV

**Figure 4:**
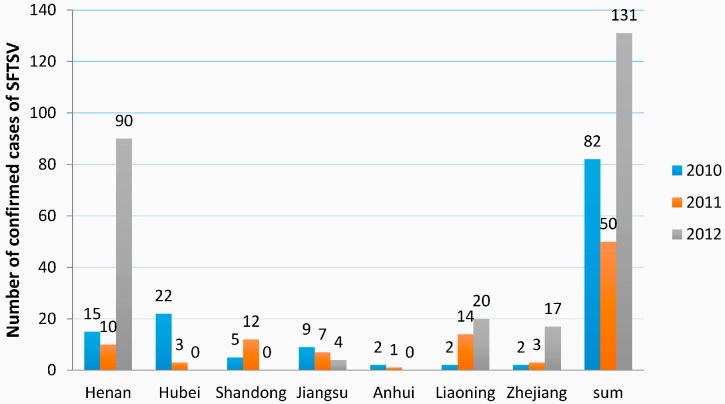
Regional distribution of the confirmed cases of SFTSV, 2010–2012. The statistics were summarized from GenBank available data until 13 March 2016. The numbers were not the actual total numbers of cases confirmed by the Ministry of Health in China.

A total of 263 confirmed cases of SFTSV (82 in 2010, 50 in 2011 and 131 in 2012) were categorized. From 2010 to 2012, the number of each province showed variable changes (Figure 4). Henan province varied with a wide range from 15 in 2010 to 90 in 2012. Hubei contained a high number of 22 in 2010 and Henan of 90 in 2012. It is worth mentioning that the frequency analysis was based on GenBank available data until 13 March in 2016 and the numbers of confirmed cased of SFTSV were not the actual total numbers of cases confirmed by the Ministry of Health in China.

### 3.3. Multicollinearity of Variables

Multicollinearity was detected using VIF scores. The findings are presented in Table 2. According to the literature, a VIF score of >10 indicates strong multicollinearity, a VIF score of between 5 and 10 indicates moderate multicollinearity and a VIF score of between 2 and 5 indicates mild multicollinearity [47,48]. In our study, the highest VIF score was 3.33 (for ARH), indicating mild multicollinearity. Therefore, we did not exclude any variables from the model.

### 3.4. Construction of Models and Measures of Model Fit

In GWR 4.0, all the explanatory variables were standardized using a *z*-transformation to ensure easier interpretation of the estimated coefficients. Additionally, a fixed bi-square kernel type was employed. The model was fitted using a golden section search to find the optimal bandwidth size, and the minimum AIC value was used as the indictor.

Multiple GWLR models and corresponding LR models (which were used for model comparisons) were constructed by multiple tests. We compared the models using the AIC and AUC values, and the most appropriate model with good distinguishing capacity between the presence and absence of SFTSV was selected. We carried out three tests for optimization. In test A,${\;\beta }_{4}\left(u_{i},v_{i}\right)$ and $\beta_{5}\left(u_{i},v_{i}\right)$ were set at zero in order to consider the relationships between the meteorological factors and SFTSV. In test B, no coefficients were set at zero. In test C, only non-meteorological factors were considered, with $\beta_{1}\left(u_{i},v_{i}\right)$, $\beta_{2}\left(u_{i},v_{i}\right)$ and $\beta_{3}\left(u_{i},v_{i}\right)$ set at zero. The GWLR model in test A had the smallest AIC value and had a moderate capacity to distinguish between the presence and absence of SFTSV (AUC = 0.77). The GWLR model in test B had the largest AIC value and had a moderate capacity to distinguish between the presence and absence of SFTSV (AUC = 0.78). The GWLR model in test C had a large AIC value and a poor capacity to distinguish between the presence and absence of SFTSV (AUC = 0.68) (Table 3).

### 3.5. Estimation of Regression Coefficients of the GWLR Models

The GWLR model with $\beta_{4}\left(u_{i},v_{i}\right)$ and $\beta_{5}\left(u_{i},v_{i}\right)$ set at zero had the smallest AIC value and a moderate capacity to distinguish between the presence and absence of SFTSV and it was therefore the most appropriate model. The estimated coefficients of this model were used in the analysis of high- and low-risk areas in mainland China. The summary statistics of the varying (local) coefficients are presented in Table 4.

**Figure 5:**
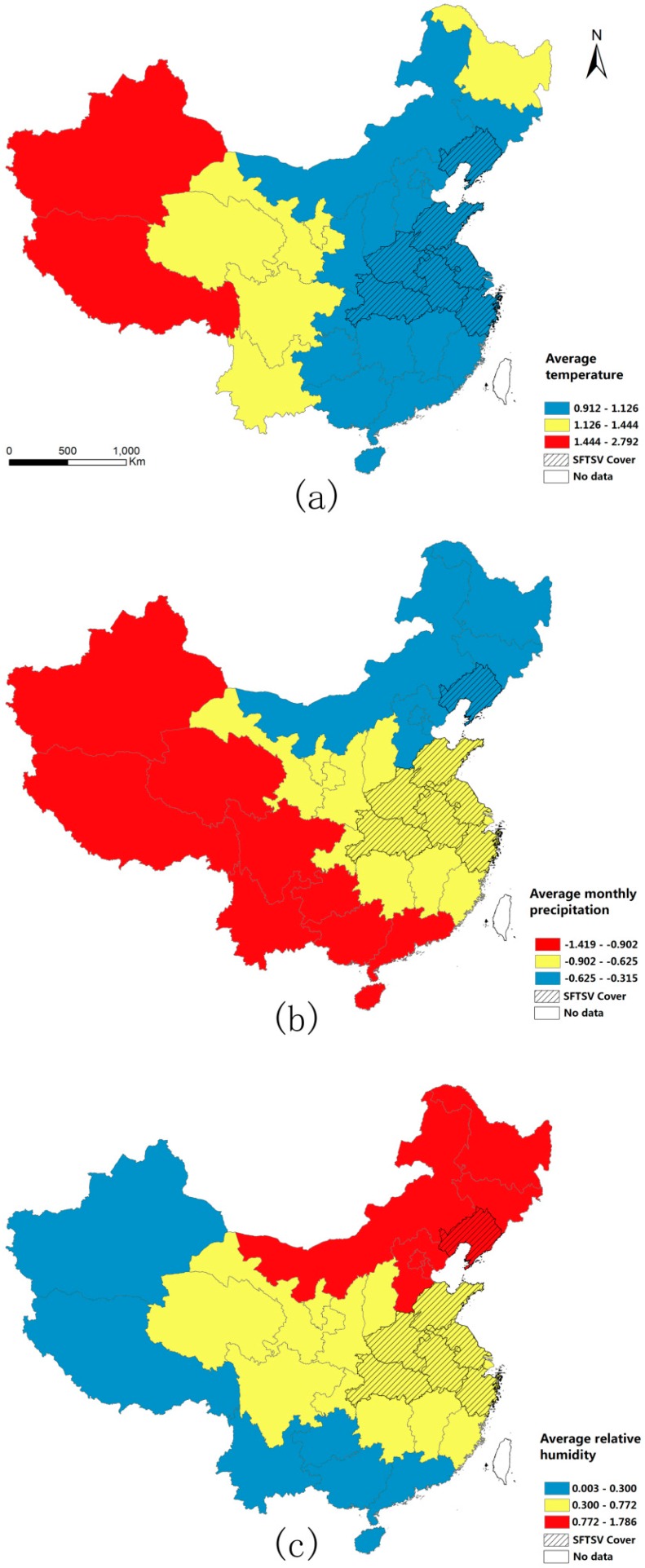
Spatial distribution of the local relationships between SFTSV cover and the three meteorological factors: (**a**) Estimated coefficient for average temperature; (**b**) Estimated coefficient for average monthly precipitation; and (**c**) Estimated coefficient for average relative humidity. SFTSV = severe fever with thrombocytopenia syndrome virus.

In the GWLR model, the sign of the estimated coefficient for each explanatory variable indicates each variable’s direction of effect on the probability of SFTSV cover. All of the explanatory variables were standardized, and the absolute values of the estimated coefficients reflect the strength of the associations between the explanatory variables and the probability of SFTSV cover (Figure 5). In order to simplify the analysis, the intensity of each variable was categorized into three groups (slight, moderate and strong) according to the Jenks natural breaks classification method (Table 5). To be specific, this classification is based on natural groupings inherent in the data. Class breaks are identified that best group similar values and that maximize the differences between classes [49].

**Figure 6:**
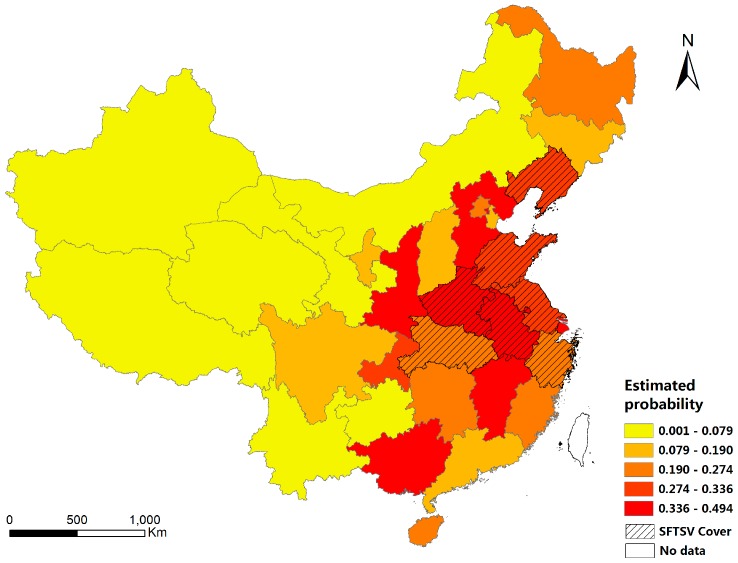
Estimated probability of SFTSV cover in each province in mainland China.

We put the values of the variables in each location into an estimation model to determine the probability of SFTSV cover. The probability of SFTSV cover was found to range from 0.001 to 0.494 (Figure 6). The eastern, central and south-central regions of mainland China had higher probabilities of SFTSV cover, while the western and mid-western regions had low probabilities of SFTSV cover.

### 3.6. Classification of Areas in Mainland China According to the Risk of SFTSV Cover Indicated by Meteorological Factors

**Figure 7:**
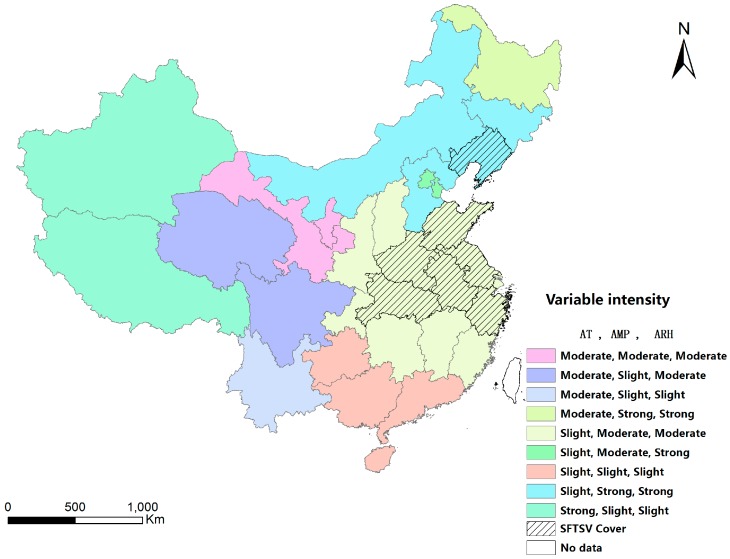
Classification of the provinces in mainland China according to the risk of SFTSV cover indicated by the three meteorological factors; AT = average temperature; AMP = average monthly precipitation; ARH = average relative humidity; SFTSV = severe fever with thrombocytopenia syndrome virus.

We overlaid the maps of the coefficients and divided the mainland into nine areas (Figure 7). We found that among the seven provinces with SFTSV cover, six provinces (Henan, Hubei, Shandong, Anhui, Jiangsu and Zhejiang) demonstrated that temperature had a slight association and precipitation and relative humidity had moderate associations, while the other province (Liaoning) demonstrated that temperature had a slight association and precipitation and relative humidity had strong associations.

In addition, 13 provinces in central China, which had warm climates and were mostly in the northern subtropical zone, were classified as having high probabilities of SFTSV cover. The values of the meteorological variables in these areas from May to July were stable from 2010 to 2012 (Table 6).

## 4. Discussion

This study found that meteorological factors (temperature, precipitation and relative humidity) were associated with SFTSV cover and that the influence of meteorological factors on SFTSV cover varies in different areas.

Numerous previous studies have explored the potential factors that influence the incidence of SFTS in various regions. One such study used a Poisson regression analysis to show that the spatial variations in the incidence of SFTS were significantly associated with shrub, forest and rain-fed cropland areas in the Xinyang region of the Henan province in China [18]. Another study conducted spatial scan statistics and multivariate model of SFTS in China using the surveillance data from 2010 to 2013, and they detected three hot spots of SFTS in China and identified independent risk factors of the distribution of SFTS [50]. Additional studies have assessed the potential factors that influence the incidence of other infectious but not vector-borne diseases, and these studies have used a variety of different methodologies. A relationship between climatic factors and local transmission was found for the 2009 pandemic influenza A virus (H1N1) in mainland China in a study that used a multilevel Poisson regression model [51]. Another study developed a logistic regression model to assess the relationships between known populations of *Ixodes scapularis* (a tick that is the main vector for Lyme disease in the USA) and environmental factors (the minimum, maximum and mean monthly temperature and vapor pressure) in order to investigate Lyme disease in the USA [25]. A third study used GWR models to explore the association of the incidence of hand, foot and mouth disease (HFMD) with child population density and climatic factors at the county level in China [52], and the authors found that child population density had a stronger influence than climatic factors on the incidence of HFMD.

In these previous studies, statistical methodologies were used to explore the factors that are associated with various infectious diseases, including climatic factors. However, they used different types of models (e.g., an OLS model, a Poisson regression model and a GWR model). An OLS model was not included in our study since SFTSV cover is binomial variable. Insignificant results would be gained if we joined binomial variable into OLS model. Another global regression model, LR model, was utilized for comparison analysis. A previous study compared a GWLR model with an orthodox LR model of the spatial variations of urban growth patterns in the Chinese city of Nanjing [35] showed that the GWLR model significantly improves on the global LR model in terms of goodness-of-fit. In the present study, we firstly estimated each GWLR model, and compared their performance with that of corresponding LR models. To be specific, three non-spatial LR models were constructed and compared with three GWLR models. The three LR models were consistent with the respective GWLR models, and the LR models had the same explanatory variables as those in the GWLR models. Our results support a growing body of recent literature [38,53,54] underlining the superiority of spatially explicit regression models relative to orthodox global models. 

Our study underlined the fact that the effect sizes of each explanatory variable in each province are potentially heterogeneous and utilized a GWLR model which is a sort of spatially explicit regression model. Additionally, this study explored both meteorological and anthropogenic factors and found that meteorological factors were associated with SFTSV cover by using GWLR models. Compared with traditional regression analysis, spatially explicit models could cover local variations and indicate spatial heterogeneity, thus underlining the notion that specific places within the same study area might differ from each other with regard to the nature and extent of meteorological injustice.

GWLR indicated that many of the observed statistical associations between SFTSV risk and specific explanatory variables are not uniform across China. More specifically, the local estimates of parameters of variables enabled us to investigate variations in the influences of the explanatory variables on SFTSV cover. From the maps of the estimated coefficients, the differences between locations regarding the strength of the associations between the explanatory variables and the probability of SFTSV cover were clear. Regarding temperature, the strength of the association decreased from east to west across China, and there was only a slight association in large areas of the mainland. Additionally, the estimated coefficients for relative humidity increased from southwest to northeast across China. The pattern for precipitation was similar to that for relative humidity; however, the coefficients increased in the opposite direction across China.

Based on our findings, we speculate that the high-risk areas are yellow areas in Figure 6, with slight temperature influence (25.5–27 °C) and moderate precipitation (442.4–644.8 mm) and moderate relative humidity (72%–80%). To be more specific in location, the areas where SFTSV has been isolated, and the provinces of Shanxi, Shaanxi, Guizhou, Hunan, Jiangxi, Fujian and Shanghai which surround the former, should be carefully monitored. Furthermore, there are several improvements that could be made to this analysis in the future. Firstly, although SFTS is a tick-borne disease, tick density was not included in the models because of the lack of data on the vector populations. We attempted to acquire data on tick density in China, but this information proved difficult to obtain. However, the model could be further improved if tick density was taken into account.

In addition, the sample size used in the analysis was small. As SFTS is a new epidemic disease, we could not obtain data on SFTS at a more granular level than province-level data. In this analysis, SFTSV data from 2010 to 2012 were obtained from the NCBI database, and province data were often included but more precise location data were rarely included. Although we only obtained approximate location data, strong factors that are associated with SFTSV cover between SFTSV and meteorological factors was found. Despite this, we recognize that this analysis is only a preliminary analysis of spatial associations and it has its limitations. The results of our study highlight the need for researchers to recognize the usefulness of GWLR as an exploratory data analysis tool for SFTSV risk assessment. More local-level research is necessary, however, to determine why statistical relationships between SFTSV cover and specific explanatory factors vary in certain parts of China. Meanwhile, we appeal to researchers to provide more precise location data when they upload SFTSV data to the NCBI database in order to aid scientific progress.

## 5. Conclusions

The study showed that a GWLR model is appropriate to assess the SFTSV cover in mainland China. Meteorological factors (temperature, precipitation and relative humidity) are associated with the SFTSV cover, and the influence of these meteorological factors on the SFTSV cover varies across different areas. Meanwhile, the provinces of Shanxi, Shaanxi, Guizhou, Hunan, Jiangxi, Fujian and Shanghai should be carefully monitored, as they surround the areas where SFTSV has been isolated and they have suitable conditions for SFTSV transmission compared with the other provinces where SFTSV has not yet been isolated.

There is currently no vaccine for SFTSV and SFTS often has fatal consequences. Our findings provide evidence to support public health policy and decision-making in allocating resources locally, which will enable better identification and detection of high risk areas, a reduction of the risk of infection and strengthened population resilience. We suggest that the efforts put into control strategies and prevention should be greater in areas where environmental factors are known to pose a significant risk. For instance, medical professionals could give extra attention to suspicious cases in high-risk areas and verify whether these cases involve SFTSV infections. Additionally, in these cases, attempts should be made to isolate the virus and to confirm the diagnosis. Further accumulation of data on cases of SFTSV infection will aid in the control of SFTSV and the prevention of SFTS.

## Figures and Tables

**Table 1:** Units of the variables.

	AT	AMP	ARH	APRP	APPI
Unit	°C	mm	%	%	%

AT = average temperature; AMP = average monthly precipitation; ARH = average relative humidity; APRP = average proportion of rural population; APPI = average proportion of primary industries.

**Table 2:** VIF scores of the variables.

	AT	AMP	ARH	APRP	APPI
VIF score	1.50	3.25	3.33	2.71	2.74

VIF = variance inflation factor; AT = average temperature; AMP = average monthly precipitation; ARH = average relative humidity; APRP = average proportion of rural population; APPI = average proportion of primary industries.

**Table 3:** Measures of model fit.

Test	A		B		C	
Model Type	LR	GWLR	LR	GWLR	LR	GWLR
AIC	37.586	37.242	39.738	39.651	38.796	38.545
AUC	0.70	0.77	0.75	0.78	0.54	0.68

AIC = Akaike information criterion; AUC = area under the relative operating characteristic curve; GWLR = geographically weighted logistic regression; LR = logistic regression.

**Table 4:** Summary statistics of the varying (local) coefficients.

Coefficient Label	Minimum	Maximum	Mean	Range	Standard Error
Intercept	−3.324	−1.218	−1.506	2.106	0.426
AT	0.912	2.792	1.133	1.880	0.363
AMP	−1.419	−0.315	−0.710	1.104	0.227
ARH	0.002	1.786	0.571	1.789	0.382

AMP = average monthly precipitation; ARH = average relative humidity; AT = average temperature.

**Table 5:** Categories of Estimated coefficients.

Coefficient Label	Slight	Moderate	Strong
AT	0.912–1.126	1.126–1.444	1.444–2.792
AMP	−1.419–−0.902	−0.902–−0.625	−0.625–−0.315
ARH	0.003–0.300	0.300–0.772	0.772–1.786

AMP = average monthly precipitation; ARH = average relative humidity; AT = average temperature.

**Table 6:** AT, AMP and ARH from 2010 to 2012 in the 13 provinces in China with high probabilities of SFTSV cover.

Year	Coefficient Label	Mean	Minimum	Maximum
2010	AT (°C)	25.2	23.3	26.8
	AMP (mm)	430.4	81.0	686.2
	ARH (%)	69.2	49.1	78.3
2011	AT (°C)	25.5	22.0	27.2
	AMP (mm)	415.3	113.5	681.2
	ARH (%)	66.5	51.7	81.0
2012	AT (°C)	25.9	22.8	27.7
	AMP (mm)	415.3	113.5	681.2
	ARH (%)	68.2	52.7	81.0

AMP = average monthly precipitation; ARH = average relative humidity; AT = average temperature.
